# Identification of phenotype-specific networks from paired gene expression–cell shape imaging data

**DOI:** 10.1101/gr.276059.121

**Published:** 2022-04

**Authors:** Charlie George Barker, Eirini Petsalaki, Girolamo Giudice, Julia Sero, Emmanuel Nsa Ekpenyong, Chris Bakal, Evangelia Petsalaki

**Affiliations:** 1 European Molecular Biology Laboratory-European Bioinformatics Institute, Hinxton CB10 1SD, United Kingdom;; 2 University of Bath, Claverton Down, Bath BA2 7AY, United Kingdom;; 3 Institute of Cancer Research, London SW3 6JB, United Kingdom

## Abstract

The morphology of breast cancer cells is often used as an indicator of tumor severity and prognosis. Additionally, morphology can be used to identify more fine-grained, molecular developments within a cancer cell, such as transcriptomic changes and signaling pathway activity. Delineating the interface between morphology and signaling is important to understand the mechanical cues that a cell processes in order to undergo epithelial-to-mesenchymal transition and consequently metastasize. However, the exact regulatory systems that define these changes remain poorly characterized. In this study, we used a network-systems approach to integrate imaging data and RNA-seq expression data. Our workflow allowed the discovery of unbiased and context-specific gene expression signatures and cell signaling subnetworks relevant to the regulation of cell shape, rather than focusing on the identification of previously known, but not always representative, pathways. By constructing a cell-shape signaling network from shape-correlated gene expression modules and their upstream regulators, we found central roles for developmental pathways such as WNT and Notch, as well as evidence for the fine control of NF-kB signaling by numerous kinase and transcriptional regulators. Further analysis of our network implicates a gene expression module enriched in the RAP1 signaling pathway as a mediator between the sensing of mechanical stimuli and regulation of NF-kB activity, with specific relevance to cell shape in breast cancer.

The study of cancer has long been associated with changes in cell shape as morphology can be a reliable way to subtype cancer and predict patient prognosis (Wu et al. 2020). Recent research has implicated cellular morphology in more than just a prognostic role in cancer, with shape affecting tumor progression through the modulation of migration, invasion, and overall tissue structure (Krakhmal et al. 2015; Baskaran et al. 2020). The unique mechanical properties of the tumor tissue (primarily driven by changes in cell shape and the extracellular matrix) are hypothesized to contribute to the “stem cell niche” of cancer cells that enables them to self-renew as they do in embryonic development (Cooper and Giancotti 2019). Cell morphology and tumor organization have been found to be a factor in modulating the intracellular signaling state through pathways able to integrate mechanical stimuli from the extracellular environment (Orsulic et al. 1999; Miralles et al. 2003; Zheng et al. 2009; Olson and Nordheim 2010). The discovery of mechanosensitive pathways in various tissues has revealed a complex interplay between cell morphology and signaling (Kumar et al. 2016). Further studies have revealed that cell morphology can also be a predictor of tumorigenic and metastatic potential as certain nuclear and cytoplasmic features enhance cell motility and spread to secondary sites (Wu et al. 2020), aided by the epithelial-to-mesenchymal transition (EMT). This process is the conversion of epithelial cells to a mesenchymal phenotype, which contributes to metastasis in cancer and to worse prognosis in patients (Roche 2018).

Breast cancer is the most common cancer among women and, in most cases, is treatable, with a survival rate of 99% among patients with a locally contained tumor. However, among those patients presenting with a metastatic tumor, this rate drops to 27% (Siegel et al. 2019). During the development of breast cancer tumors, cells undergo progressive transcriptional and morphological changes that can ultimately lead toward EMT and subsequent metastasis (Lee et al. 2015b; Feng et al. 2018; Wu et al. 2020). Breast cancer subtypes of distinct shapes show differing capacities to undergo this transition. For example, long and protrusive basal breast cancer cell lines are more susceptible to EMT (Fedele et al. 2017) with fewer cell-to-cell contacts (Dai et al. 2015). Luminal tumor subtypes, on the other hand, are associated with good to intermediate outcomes for patients (Dai et al. 2015) and have a clear epithelial (or “cobblestone”) morphology with increased cell–cell contacts (Neve et al. 2006). It is evident that cell morphology plays significant roles in breast cancer, and a deeper understanding of the underlying mechanisms may offer possibilities for using these morphology-determinant pathways as potential therapeutic targets and predictors of prognosis.

Signaling and transcriptomic programs are known to be modulated by external physical cues in the contexts of embryonic development (Wozniak and Chen 2009), stem-cell maintenance (Bergert et al. 2021; De Belly et al. 2021), and angiogenesis (Chatterjee 2018). Numerous studies have flagged NF-kB as a focal point for mechanotransductive pathways in various contents (Cowell et al. 2009; Shrum et al. 2009; Tong and Tergaonkar 2014; Ishihara et al. 2019), but gaps in our knowledge remain as to how these pathways may interact and affect breast cancer development. Sero et al. (2015) studied the link between cell shape in breast cancer and NF-kB activation by combining high-throughput image analysis of breast cancer cell lines with network modeling. They found a relationship between cell shape, mechanical stimuli, and cellular responses to NF-kB and hypothesized that this generated a negative feedback loop, in which a mesenchymal-related morphology enables a cell to become more susceptible to EMT, thus reinforcing their metastatic fate. This analysis was extended by Sailem and Bakal (2017), who combined cell-shape features collected from image analysis with microarray expression data for breast cancer cell lines to create a shape–gene interaction network that better delineated the nature of NF-kB regulation by cell shape in breast cancer. This approach was limited, as it only correlates single genes with cell shapes, thus relying on the assumption that a gene's expression is always a useful indicator of its activity (Vogel and Marcotte 2012). Furthermore, the investigators rely on a list of preselected transcription factors (TFs) of interest, and as such, the approach is not completely data-driven and hypothesis-free. Given our knowledge of the multitude of complex interacting signaling pathways in development and other contexts, it is safe to assume that there are many more players in the regulation of cancer cell morphology that have yet to be delineated (Bougault et al. 2012; Rolfe et al. 2014; Robertson et al. 2015; Horton et al. 2016). Furthermore, how exactly extracellular mechanical cues are “sensed” by the cell and passed on to NF-kB in breast cancer is not clearly understood. From this it is clear that an unbiased approach is needed to identify novel roles for proteins in the interaction between cell shape and signaling.

Here we have developed a powerful network-based approach to bridge the gap between widely available and cheap expression data, signaling events, and large-scale biological phenotypes such as cell shape (Fig. 1A). Our study aims to identify a data-derived cellular signaling network, specific to the regulation of cell shape beyond NF-kB, by considering functional coexpression modules and cell signaling processes rather than individual genes.

**Figure 1.**
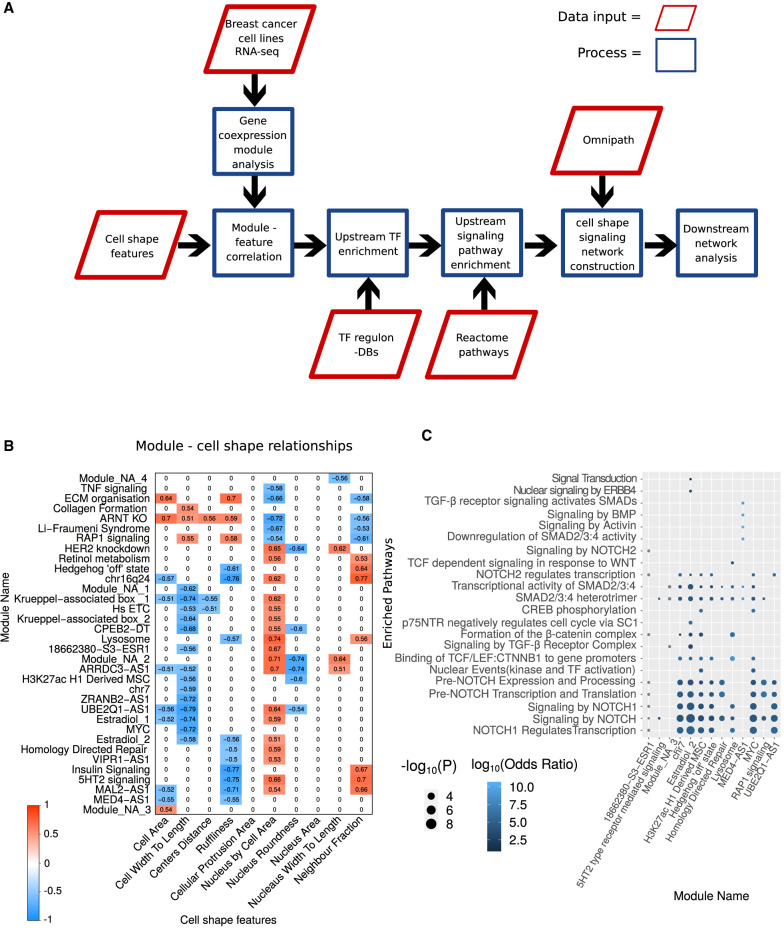
Overview of workflow and resultant gene expression modules and pathways. (*A*) Schematic illustrating the steps involved in phenotype-specific network construction. Gene expression modules are identified by integrating cell-shape variables (derived from imaging data) with RNA-seq data from breast cancer cell lines. These gene expression modules are correlated with specific cell-shape features to find morphologically relevant modules. Next, transcription factors (TFs) are identified whose targets significantly overlap with the contents of the expression modules. These TFs are used to identify pathways regulating the gene expression modules, which are then integrated to form a contiguous network using PCSF. (*B*) Heat map of significantly correlated gene expression module eigengenes with cell-shape features. Non significant interactions were set to zero for clarity. (*C*) Dot plot illustrating the enrichment of pathways among TFs found to regulate gene expression modules. The *x*-axis shows the module names (as defined by Supplemental Table S3), and the *y*-axis shows the signaling pathways found to be significantly (*P* < 0.01) enriched in the TFs that regulate the given module (as defined by Supplemental Table S5). The *y*-axis is arranged such that the terms with the highest combined odds ratio are at the *bottom*. Size of the dot represents the −log_10_(P), and the color indicates a log_10_ transformation of the odds ratio.

## Results

### Identification of gene coexpression modules correlated with cell-shape features

We first sought to identify gene expression modules (GEMs) that are relevant to the regulation of cell shape. To this end, we used weighted gene correlation network analysis (WGCNA) (Langfelder and Horvath 2008) on bulk RNA-seq expression data from 13 breast cancer cell lines and one nontumorigenic epithelial breast cell line to identify gene coexpression modules correlated with 10 specific cell-shape variables (Methods; Sailem and Bakal 2017). These described the size, perimeter, and texture of the cell and the nucleus (*n* = 75,653). Of 102 GEMs (Supplemental Fig. S1A), 34 were significantly correlated (*P* < 0.05; Student's *t*-test, Pearson's correlation) (Supplemental Table S1; Supplemental Fig. S1B,C) with one of eight cell-shape features (Fig. 1B). A full list of the genes within the identified modules is presented in Supplemental Table S2.

We used Enrichr and their suite of gene set libraries (Kuleshov et al. 2016) to functionally annotate and label some of the modules using enrichment of genes contained within them. We found that the “RAP1 signaling” module is also enriched for terms such as VEGF signaling and hemostasis, whereas the “insulin signaling” module is also enriched for cell–cell communication, and the “ECM organization” module is also enriched in terms such as axon guidance and EPH–ephrin signaling (Supplemental Table S3). Modules that are most correlated with all features are the “*ARNT* KO” module, “ARRDC3−AS1” module, and the “ECM organization” module (Supplemental Fig. S1B). Modules that could not be annotated with informative terms were designated as module “non annotated” (NA) 1, 2, 3, etc.

### TF analysis of cell-shape gene coexpression modules reveals the signaling pathways that regulate them

To link these expression modules to the intracellular signaling network, we considered both the regulation of modules as transcriptional units as well as the signaling pathways that significantly regulate the identified regulons. Specifically, we first found 17 TF regulons, as defined in the database TRRUST v2 (Han et al. 2018), to be significantly enriched (*P* < 0.1; Fisher's exact test) in our modules (Supplemental Table S4). We therefore consider these TFs as potentially relevant for the regulation of cell-shape features and their activity levels as a read-out of cell signaling activity in these cells. These TFs include the EMT antagonist FOXA1 (Song et al. 2010), as well as HOXB7 (Wu et al. 2006) and ZFP36 (Van Tubergen et al. 2013).

To extend this further, we sought to investigate the pathways responsible for regulating the identified TFs and, by extension, the GEMs. For this analysis, we also include ENCODE and ChEA Consensus TFs from ChIP-X (Lachmann et al. 2010), DNA binding preferences from JASPAR (Wasserman and Sandelin 2004; Stormo 2013), TF protein–protein interactions, and TFs from ENCODE ChIP-seq (Euskirchen et al. 2007) to get a more comprehensive picture of the pathways involved in regulation of cell morphology. Using the identified TFs (Supplemental Table S5) we then used Enrichr (Kuleshov et al. 2016) to perform a Reactome signaling pathway (Jassal et al. 2020) enrichment analysis. Results from this analysis showed that six modules shared pathways associated with downstream signaling and regulation of NOTCH (Fig. 1C). To ensure that our approach is not biased to any particular pathway, we repeated our approach on 1000 resampled GEMs, and created pathway-specific null distributions for each identified pathway. All pathways we identified from morphology-correlated modules had significantly lower *P*-values than randomized modules (FDR adjusted *P* < 0.05). The only exceptions were one association with “signaling by NOTCH” and modules associated with “signal transduction,” a spurious pathway containing the complete intracellular signaling system (Supplemental Table S6).

### Clustering based on morphology reveals distinctive cell line shapes

**Figure 2.**
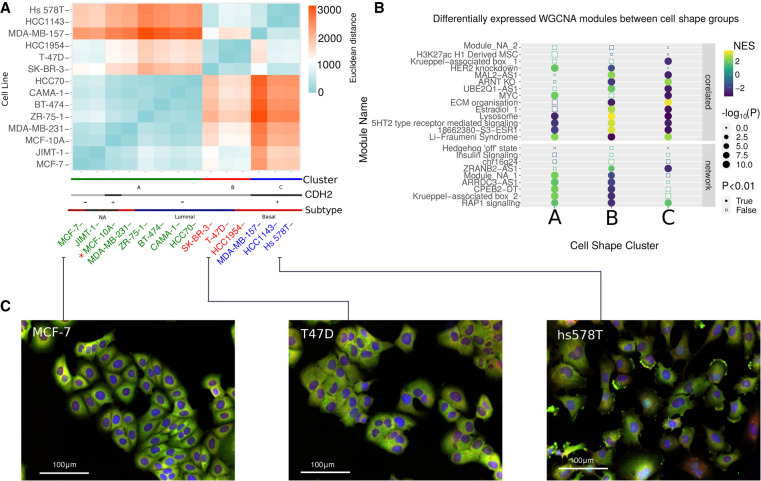
Clustering breast cancer cell lines into groups of similar morphology. (*A*) Heat map of Euclidean distance between cell lines for shape features to illustrate clusters arising from *k*-means method. The colored lines on the *bottom* show the assigned cluster and the cadherin expression and assigned canonical cancer subtype. (*B*) Dot plot showing the enrichment of gene expression modules in the different cell line clusters. Along the *y*-axis are the names of the clusters, faceted by whether they are included in the PCSF-derived regulatory network on the *bottom* and whether they are correlated with cell-shape variables, but not included in the network on the *top*. The *x*-axis shows the cell-shape clusters, with letters corresponding to the groups in *A*. (A) A heterogeneous mix of breast cancer subtypes, (B) luminal-like cell lines, (C) basal-like cell lines. Dots are colored based on the normalized enrichment value, with down-regulated modules in blue and the up-regulated modules in yellow. Size corresponds to significance [−log_10_(P)] with the shape illustrating which changes are significant (adjusted *P* < 0.01, Benjamini–Hochberg). (*C*) Images (see Methods) showing morphology of representative cell lines from each respective cluster. Colors indicate labeling with DAPI (blue), anti-p65 (green), and DHE (red).

To understand key differences in expression patterns and gene regulation between morphologically distinct breast cancer cell lines, we clustered them based on 10 morphological features including area, ruffliness, protrusion area, and neighbor frequency and performed differential expression analysis between the identified clusters (Fig. 2A; Supplemental Fig. S2A). Cluster A is more heterogeneous in its morphology, containing the nontumorigenic mammary epithelial cell line MCF-10A as well as cell lines from both luminal and basal breast cancer subtypes. Clusters B and C are more distinctly shaped, roughly composed of luminal and basal cell lines, respectively, except for HCC1954, which was clustered morphologically with luminal subtypes, while being characterized as basal. The basal-like cluster is most morphologically distinct from cluster A but also differs from the luminal-like cluster in that it has a lower nuclear/cytoplasmic area (0.133 ± 0.05 [mean ± SD]), higher ruffliness (0.235 ± 0.12), and lower neighbor fraction (0.258 ± 0.22). The luminal-like cluster had a higher nuclear/cytoplasmic area (0.186 ± 0.1; *P* < 0.001), lower ruffliness (0.213 ± 0.14; *P* < 0.001), and a higher neighbor fraction (0.338 ± 0.26; *P* < 0.001, one-way ANOVA; Tukey HS, n = 75,653). The neighbor fraction feature corresponds to the fraction of the cell membrane that is in contact with neighboring cells. The lower number of cell–cell contacts in basal-like breast cancer cell lines is indicative of more mesenchymal features associated with worse prognosis owing to metastasis. Increased cell–cell contacts in both the luminal-like cluster and the more heterogeneous cluster A correspond to “cobblestone” epithelial morphology. We found that these groups are closely aligned with the expression of the cell adhesion protein cadherin 2 (CDH2, also known as N-cadherin) (Fig. 2A), the expression for which is closely associated with a migratory and metastatic phenotype (Shih and Yamada 2012). Representative images of the morphologically clustered cell lines are shown along with the clustering heat map in Figure 2A (complete data set of images provided online; https://datadryad.org/stash/dataset/doi:10.5061/dryad.tc5g4).

Using the identified groups of cell lines in the previous step, differential expression analysis and TF activity analysis were used to study gene regulation signatures specific to cell line morphological clusters. The results are shown in Supplemental Table S7, with gene set enrichment analysis showing up-regulation of genes involved in the extracellular matrix, collagens, integrins, and angiogenesis in the basal-like cluster. Significantly enriched terms (*P* < 0.05) in down-regulated genes include “fatty acid and beta-oxidation” and “ERBB network pathway.” In the genes up-regulated in the luminal-like cluster, we observed enrichment of terms such as “hallmark-oxidative phosphorylation,” down-regulated genes were enriched in “integrin-1 pathway,” “core matrisome,” and genes linked to “hallmark epithelial–mesenchymal transition and migration.” For the remaining B/L group, the term with the highest normalized enrichment score was “targets of the transcription factor MYC” followed by terms associated with ribosomal RNA processing. Down-regulated terms include “cadherin signaling pathway” (Supplemental Table S7).

We also calculated the differential expression for the WGCNA GEMs and found distinct patterns of expression between luminal-like and basal-like clusters of cell lines (Fig. 2B). Among these, the RAP1 signaling module is up-regulated in basal-like clusters and down-regulated in luminal-like clusters. This is consistent with the fact that this GEM is negatively correlated with neighbor fraction, a feature that is observed to decrease in mesenchymal-like cell shapes (Dai et al. 2015). Other modules whose expression distinguishes basal-like from luminal-like include the MAL2-AS1 module (enriched in desmosome assembly), *ARNT*/KO module (enriched in TNF-signaling by NF-kB), and ECM organization module (enriched in focal adhesion proteins) (see Supplemental Table S3).

To link the observed gene expression differences (Supplemental Fig. S2B,C) to cell signaling, we used the tool DOROTHEA (Garcia-Alonso et al. 2019) to calculate TF activities, as their modulation is one of the main results of cell signaling processes. We corroborated that the heterogenous B/L group had significantly activated MYC levels. In the luminal-like cluster, estrogen-related receptor alpha (ESRRA) is the most significantly overrepresented regulome, followed by EHF, KLF5, and ZEB2. Underrepresented regulomes include KLF4, SMAD4, SMAD2, SOX2, and RUNX2. For the basal-like cluster, the regulome with the highest normalized enrichment score is SOX2, as well as MSC and HOXA9. Down-regulated regulomes include ZEB2, MYC, ESRRA, and KLF5 (Supplemental Fig. S2D).

### Assembly of a data-driven cell-shape regulatory network

To integrate our data-driven GEMs with signaling pathways, we used the prize-collecting Steiner forest (PCSF) algorithm (Akhmedov et al. 2016). This is an approach that aims to maximize the collection of “prizes” associated with inclusion of relevant nodes, while minimizing the costs associated with edge-weights in a network. This allowed for the integration of the WGCNA modules, the Reactome pathways that regulate them, the TRRUST TFs, and the differentially expressed DOROTHEA regulons into a contiguous regulatory network describing the interplay between cell-shape and breast cancer signaling. The network used for this process was extracted from the database OmniPath (Türei et al. 2016) to provide a map of the intracellular signaling network described as a signed and directed graph. We incorporated identified GEMs into the network by interlinking them as nodes with the relevant TFs and signaling pathways.

The resulting network of 691 nodes included 97.11% of the genes identified by our analysis. The new proteins that were included by the PCSF algorithm to maximize prize collection showed gene set enrichment of common terms (Pathways from PANTHER) (Mi et al. 2013) relative to the original prizes (including WNT, EGF, angiogenesis, RAS, cadherin, and TGFB pathways) but also included are some new terms (VEGF, integrin, and endothelin pathways; *P* < 0.001) (Supplemental Fig. S3A).

Studying the network properties of our PCSF-derived regulatory network, we found that the degree distribution is typical for a biological network (Supplemental Fig. S3B–D). The proteins in the network can be ranked by betweenness centrality to disseminate them based on network importance. Nodes with high centrality lie between many paths and can control information flow. Proteins with the highest centrality are primarily prizes (GSK3B, ESR1, TP53, SMAD3) (Supplemental Fig. S3E), indicating that the PCSF solution was not achieved by the inclusion of new hub proteins that are not of interest to our analysis. Nevertheless, a small minority of high centrality nodes were not in the original prizes, implicating them as mediating the cross talk between pathways identified in Figure 1C. These include the proteins PAX7, PTEN, and PPARGC1A.

### Small-molecule inhibitors targeting kinases in our network significantly perturb cell morphology

To validate our network, we used an independent data set to evaluate whether perturbing the function of kinases within our predicted network would produce a significant effect on morphological features. For this, we used the Broad Institute's Library of Integrated Network-Based Cellular Signatures’ (LINCS) small-molecule kinase inhibitor data set (Subramanian et al. 2017). Here, they measured morphological changes in the breast cancer cell line Hs 578T in response to various small-molecule kinase inhibitors using high-throughput imaging techniques (Hamilton et al. 2007). The morphological variables measured in this data set are mostly analogous to the ones used to construct the network; however, there are some discrepancies that we used as negative controls to ensure our network was phenotype-specific.

**Figure 3.**
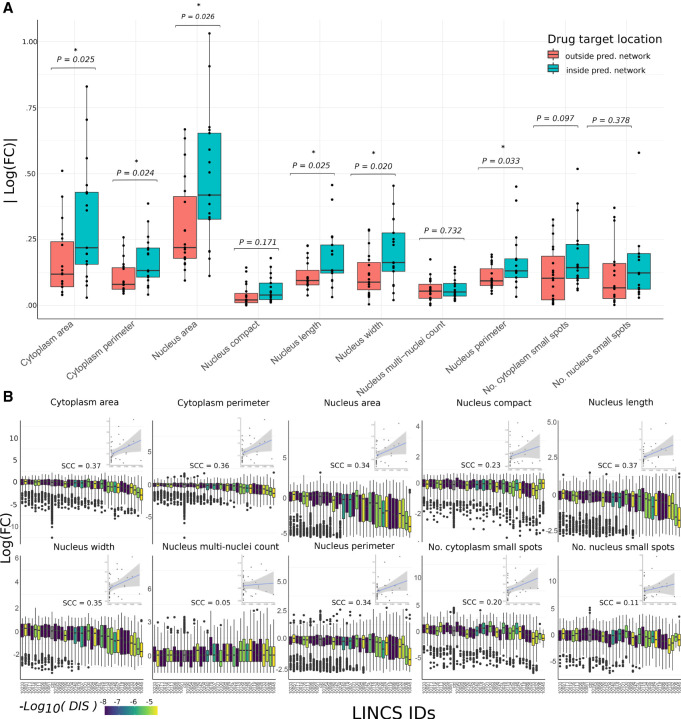
Effect of drug perturbation of derived network on breast cancer cell line morphology. (*A*) Box plots showing the absolute log_10_ fold changes after treatment with a drug relative to a control for each cell-shape variable. The drugs are grouped by those targeting kinases within the predicted regulatory network (blue) and those targeting other kinases not predicted to be associated with cell shape (red). *P*-values (Welch two-sample *t*-test) are shown with asterisks indicating significance. (*B*) Bar plot showing the absolute difference in log fold changes of cell-shape variables after treatment with a drug relative to a control. Here, each drug is shown separately (with the LINCs ID shown on the *x*-axis) and colored based on the drug influence score (DIS), and each data point represents a single cell. *Insets* are plots showing the correlation between this influence score and the difference between mean treated cells and mean control cells in each of the 10 measured cell-shape features for each drug. Spearman's correlation coefficients are shown *above* the *inset* plots.

We combined this with data from a target affinity assay (Moret et al. 2019) describing the binding affinities of small molecules to kinases. This enabled us to sort the kinase inhibitors into those that target proteins we predict regulate cell shape (through their inclusion in the PCSF-derived network) and those that do not. We found that there is a statistically significant (*n* = 37, Wald test *P* < 0.05) deviation from the control between drug treatments targeting kinases within the predicted network and those targeting other proteins for cytoplasmic area, cytoplasmic perimeter, nucleus area, nucleus length, nucleus width, and nucleus perimeter (Fig. 3A; Supplemental Fig. S4A). This difference is insignificant for features that were not correlated with gene expression modules in our initial analysis (such as number of small spots in the cytoplasm and nucleus, as well as nuclear compactness), indicating that our network is phenotype-specific to the features used in network generation. We also repeated this analysis in other cell lines (SK-BR-3, MCF-7, and nontumorigenic mammary cell line MCF-10A) with results with limited statistical significance (Supplemental Table S8). We additionally used a positive control in which the control cells had been treated with TNF-related apoptosis-inducing ligand (TRAIL) to ensure that the observed morphological effects were not caused by apoptotic factors (Supplemental Fig. S4B; Supplemental Table S8).

We found that there is greater variance in the effect size for kinase inhibitors targeting proteins contained within the predicted regulatory network than those outside. The individual effect on cell morphology for each drug is shown in Supplemental Figure S4, A and B. We hypothesized that it was the network properties of kinases within our network that dictated their effect on morphological features, with some targets being on the periphery of our predicted network and therefore having limited influence over the regulation of cell shape. To test this, we studied the extent to which the effect of a kinase inhibitor was correlated with the combined centrality of its targets as defined by our network. For this we used the centrality algorithm PageRank (Brin and Page 1998) and accounted for off-target effects of the kinase inhibitors using the Szymkiewicz–Simpson index (describing the overlap of a kinase inhibitor's targets and the proteins that constitute the network) (Methods).

Figure 3B shows moderate correlations between target centrality and the effect size for each feature, illustrating that kinase inhibitors targeting proteins with high centrality in our network modulate cell shape more than inhibitors with peripheral targets. As with studying the effect of targeting kinases contained within our network versus those outside of it, this correlation is higher among morphological variables that are the same or similar to those cell-shape features correlated with GEMs used to construct the network. The correlation between combined centrality and drug absolute effect on cell area (*n* = 37) was moderate but significant for cytoplasm area, cytoplasm perimeter, nucleus area, nucleus length, nucleus half-width, and nucleus perimeter (with Spearman's correlation coefficient between 0.34–0.37 for all of them, with *P* < 0.05). This correlation in change in morphological features with the centrality of the targeted kinases illustrates the relevance of our constructed network in regulating cell shape. For variables that were not correlated to any GEM, we see visibly lower correlation coefficients and insignificant associations (Spearman's correlation coefficients of 0.05–0.29, *P* > 0.05). These results illustrate that the topology of our network explains some of the variation in the effect of kinase inhibitors tested in a manner that is feature specific to the ones that were used to construct the network model.

### Network propagation of activated TFs reveals differentially activated processes in the cell-shape regulatory network

**Figure 4.**
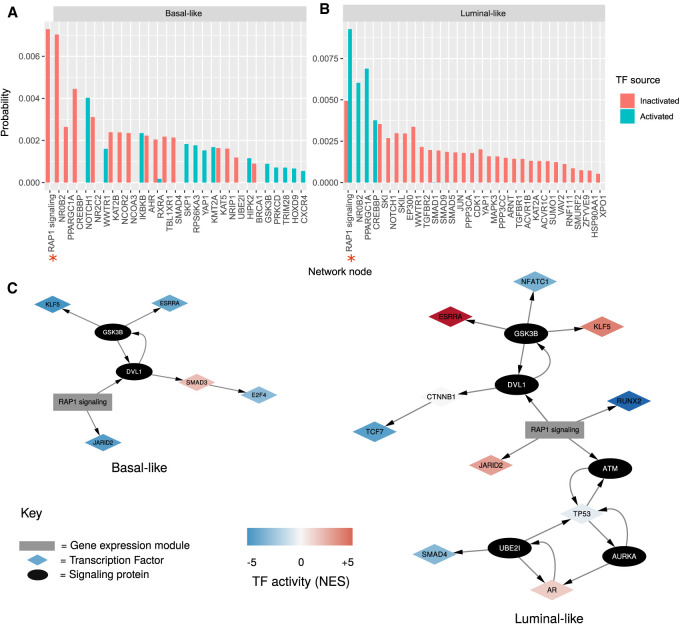
Network propagation of active TFs within cell-shape network. (*A*,*B*) Bar plot showing network propagation in a predicted cell-shape network from activated and inactivated TFs in basal-like cell lines (*A*) and luminal (*B*). The *y*-axis is a steady-state probability (or the “heat” of the nodes in the network after the diffusion) over the graph imposed by the starting seeds, ordered by size. Red bars represent propagation from TF seeds that are predicted to be inactivated, and blue bars show propagation from TF seeds that are predicted to be activated. Red asterisks along the *x*-axis indicate supernodes that represent gene expression modules. Only those nodes with combined probability > 0.0001 are shown, with the full results available in Supplemental Table S9. (*C*) Subnetworks illustrating the paths between activated TFs (in basal-like and luminal-like) and the “RAP1 signaling” gene expression module. TFs are shown as diamond-shaped nodes, with their color representing their activity. The “RAP1 signaling” gene expression module is shown as a gray rectangle. Signaling proteins are shown as black nodes.

As TF activity remains the most reliable indicator of signaling that can be extracted from transcriptomics data (Szalai and Saez-Rodriguez 2020), we applied network propagation to identify subnetworks and nodes of which differentially regulated TFs have an effect. The algorithm random walk with restart (RWR) (Tong et al. 2008) was used to diffuse from activated and inactivated TFs in our network reflected by the normalized enrichment scores of TFs identified by DOROTHEA (Methods; Fig. 4A,B; Supplemental Table S9; Garcia-Alonso et al. 2019).

The most relevant supernode in both luminal and basal diffusions was the GEM, RAP1 signaling, a module that is correlated with several cell-shape variables (neighbor frequency, ruffliness, nuclear by cytosolic area, and cell width to length) and is enriched in members of the mechanosensitive RAP1 signaling pathway. By performing RWR diffusions on each of the seed nodes separately (Supplemental Fig. S5A,B), we can see that the source of this module's probability is mainly from the TFs JARID2 and RUNX2 in luminal-like cell lines and from JARID2 for basal-like. However, the TFs KLF5 and ESRRA in both morphological subtypes also contribute to the ranking of RAP1 signaling via GSK3B and DVL1 (Fig. 4C).

Specific proteins that were top ranked after performing the network propagation in basal-like cell lines include the orphan nuclear receptor NR0B2. Individual RWR found three seed TFs responsible for this node's high probability: AR, ESRRA, and NR1H3. Other proteins flagged by the propagation were SMAD4, which is regulated by TGFB; IKBKB, which is an activator of NF-kB; and YAP1. For luminal-like cell lines, NR0B2 is also significantly ranked from the network propagation (as a result of ESRRA activity), as well as transcriptional coactivator PPARGC1A and CREBBP.

### The RAP1 GEM correlates with known morphologically relevant TFs in both cell culture and clinical samples

**Figure 5.**
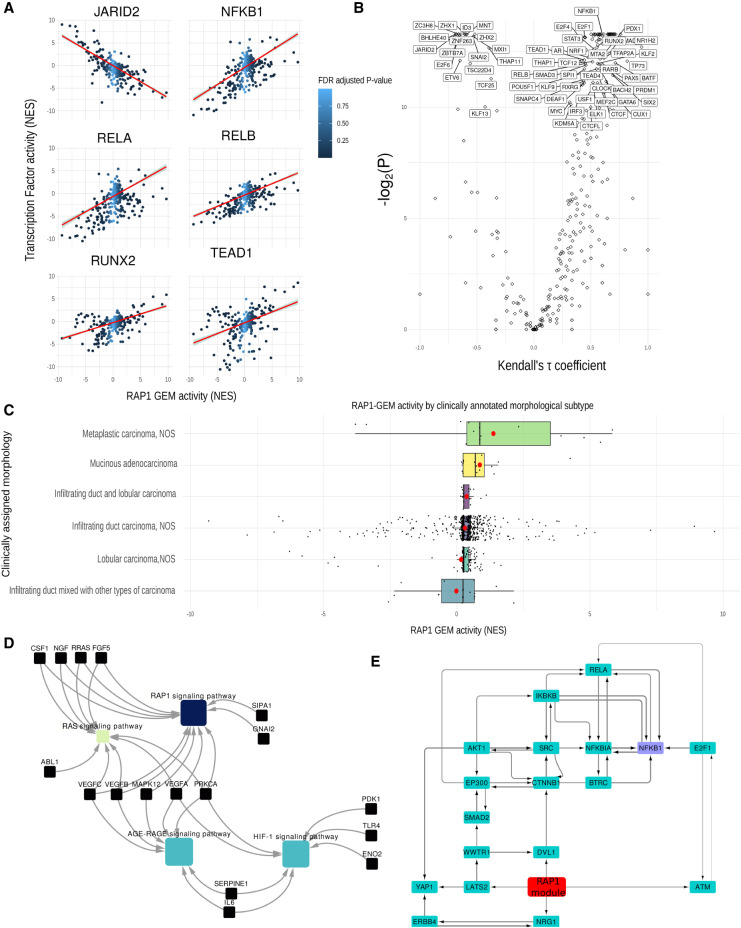
Expression of RAP1 gene expression module in further breast cancer cell lines and in clinical samples. (*A*) Plots showing the correlation between the RAP1 gene expression module activity (normalized enrichment score) (see Methods) and the activity (NES) of various TF (JARID2, NF-kB1, RELA, RELB, RUNX2, and TEAD1). The line of best fit according to linear regression is shown in red, with the confidence interval in gray. Color of the points in the plot represents the FDR adjusted *P*-value of the RAP1 NES as calculated by DOROTHEA. (*B*) Volcano plot illustrating the correlation (Kendall's rank correlation) between activity of RAP1 gene expression module and TF activity, with Kendall's tau coefficient along the *x*-axis and -log_2_(FDR adjusted *P*) along the *y*-axis. (*C*) Bar plot showing RAP1-GEM activity across different breast cancer samples, separated by clinically assigned morphology. The *y*-axis shows RAP1 gene expression module activity as calculated by DOROTHEA in NES. Mean values for each group are shown by a red dot. (*D*) Network showing gene set enrichments of the contents of RAP1 gene expression module. Genes are shown in pale blue, and pathways are shown by nodes whose color indicates significance of the associated term [−log_2_(*P*)]. (*E*) Subnetwork showing the top flow-carrying edges (99th percentile) calculated using the maximum-flow algorithm between RAP1 gene expression module and NFKB1.

To explore the significance of the RAP1 GEM in breast cancer, we measured its activity (Methods) in 78 BRCA cell lines. This enabled the correlation of its combined activity with the activity of known TFs predicted by DOROTHEA (Supplemental Fig. S6; Holland et al. 2020). We found that RAP1 GEM activity was significantly correlated (Kendall; *P* < 0.01, FDR adjusted) with the activity of 19 TFs. Among these are RUNX2 (consistent with the results from our network propagation), TEAD1 (TF mediating the function of YAP1/TAZ), and NFKB1. We also correlated TF activity using the same method on tumor samples extracted from The Cancer Genome Atlas (TCGA; https://www.cancer.gov/tcga). Using this publicly available data set, we studied 1090 BRCA tumors and performed differential expression on each sample. We found 40 TFs significantly correlated (Kendall; *P* < 0.01, FDR adjusted) with RAP1 GEM (Fig. 5A,B). The intersection of this analysis between in cell lines and the clinical data were the TFs: SP3, NFKB1, ZNF589, ZC3H8, HIF1A, STAT1, ZNF584, ZNF175, and KLF5.

We studied the expression of the module in tumor samples and compared different groups of clinically annotated morphological subtypes. The morphological subtype with the highest overall RAP1-GEM activity was metaplastic carcinoma, a subtype characterized by poorly cohesive sheets (Schwartz et al. 2013) and a high propensity to metastasize (Fig. 5C; Reddy et al. 2020). This morphological subtype has a distribution significantly greater (*P* < 0.005; two-sample Kolmogorov–Smirnov test) than the most frequently assigned morphological subtype (infiltrating duct carcinoma, NOS). This subtype is a common and homogenous breast cancer grouping characterized by its failure to exhibit morphological features that might allow it to be classified as anything more specific (Makki 2015).

### Content of RAP1 GEM and its network neighborhood shed light on signaling events relevant in the regulation of cell shape

To understand latent processes driven by components within our GEM, we also studied interactions between the Gene Ontology (GO) terms enriched within RAP1 GEM (Fig. 5D). This revealed that, as well as RAP1 signaling, the GEM is enriched in the AGE-RAGE signaling pathway and HIF1 signaling pathway (consistent with HIF1A's activity correlating highly with RAP1 GEM in both cell line patient data). HIF1A is known to be regulated downstream from RAP1 (Menon et al. 2012; Li et al. 2021), although not explicitly in breast cancer.

NF-kB has been previously linked to the regulation of cell shape in breast cancer. To explore the interface of RAP1 GEM with NF-kB in terms of intracellular signaling, we identified a subnetwork of our network responsible for mediating “information flow” between those two nodes using the algorithm maximum flow (Fig. 5E). By studying the flow of information from RAP1 signaling, we can see that a LATS2/WWTR1/DVL1 (all of WNT signaling) lies between the target and source nodes with much of the flow being carried via these edges. This implicates YAP1/TAZ as being a key effector of the identified GEM. This finding is supported by TEAD1 (mediating gene expression of YAP1 and WWTR1/TAZ) being among the most highly correlated of TFs with RAP1 GEM (Fig. 5B; Supplemental Fig. S6A).

## Discussion

We present a method that uses transcriptomics and phenotypic data to derive a concise subnetwork describing the signaling involved in the regulation of cell shape. This analysis recovered known processes like “adherens junction proteins,” “cadherin” (Eslami Amirabadi et al. 2019), and “integrin” (Taherian et al. 2011; Filer and Buckley 2013), as well as pathways responsible for the regulation of cell shape in development, such as WNT (Wildwater et al. 2011; Kadzik et al. 2014), TGFB (Lee et al. 2013), and NOTCH (Kontomanolis et al. 2018). All of these pathways have previously been linked to the development of metastatic phenotypes in breast cancer cells (Imamura et al. 2012; Kontomanolis et al. 2018; Yin et al. 2018). Moreover, individual TFs identified include the known promoters of metastasis: SOX2 (Liu et al. 2018, 2; Li et al. 2019, 2), HOXA9 (Ko and Naora 2014), and ESRRA (Berman et al. 2017). Also, among these TFs were known regulators of cell shape and EMT, including KLF5 (Chen et al. 2015), ZEB2 (DaSilva-Arnold et al. 2019), and MYC (Cowling and Cole 2007; Lourenco et al. 2019).

Importantly, this analysis also sheds light on processes with less-characterized associations with cell shape in cancer. We found that a GEM enriched in RAP1 signaling is significantly correlated with cell shape and is the most differentially expressed module between luminal-like and basal-like cell line clusters. We found that it was up-regulated in basal-like cell lines and down-regulated in luminal, consistent with its negative correlation with neighbor fraction, a cell-shape feature most contributing to the “cobblestone”-like features of an epithelial and nonmetastatic cell type. This GEM was also an important node in our identified signaling network, being at the network confluence of multiple activated TFs. We also showed this GEM to be expressed in patient data, with its activity being correlated with known developmental and morphologically related TFs, as well as those used to identify it in the network propagation analysis. In this way, our methodology uses cell line data for network construction and validation, but through our network approach, we focus on more general effects that can be tested and successfully validated in a wider breast cancer clinical context. Hence, we believe these results to be relevant in more general breast cancer applications but are also reflecting the inherent context specificity that exists in biology.

The namesake of our identified module, RAP1, is a small GTPase in the RAS-related protein family that has been shown to be involved in the regulation of cell adhesion and migration (Boettner and Van Aelst 2009; Zhang et al. 2017). Specifically, RAP1 has been shown to modulate and activate NF-kB activity in response to TNF stimulation in mesenchymal stem cells (Zhang et al. 2015) and to modulate migration and adhesion (Sawant et al. 2018). RAP1 is able to regulate IκB kinases (IKKs) in a spatiotemporal manner (Ohba et al. 2003) and is crucial for IKBK to be able to phosphorylate the NF-kB subunit RELA to make it competent (Teo et al. 2010). Here, we used our network-centric methodology to highlight a transcriptomic module, characterized in part by RAP1 signaling, and this is a key node in our phenotype-specific signaling network. It is possible that our observations of the significance of RAP1 are as a result of a more “direct” interaction between RAP1 and the cytoskeleton. However, the transcriptomic module that we observed accounts for a much larger system-wide rewiring than simply the modulation of cytoskeletal proteins. This implies more complex transcriptional changes that are characteristic of a more robust breast cancer niche.

The RAP1 signaling GEM identified in the network analysis represents a subset of the transcriptome observable among our analyzed cell lines. Although it is enriched in RAP1 signaling, it is important to note that it represents a collection of latent biological processes rather than a single pathway assigned to it by gene set enrichment. From our network analysis, we hypothesize that it is able to interact with intracellular signaling pathways in order to modulate TF activity and consequently cell shape. Other pathways enriched in the expression module include the HIF1 signaling pathway, which is known to be activated by RAP1 in melanoma (Lee et al. 2015a), but this has not been shown in breast cancer. Hypoxia inducible factor 1 (HIF1) is also of special relevance in tumorigenesis because hypoxia is one of the key stimuli that a cancer cell is able to process in order to determine its fate and maintain the cancer stem cell niche (Plaks et al. 2015). AGE-RAGE signaling was also enriched in our module of interest. The AGE-RAGE signaling pathway has recently been shown to overlap with the RAP1 signaling pathway in cardiac fibroblasts to alter the expression of NF-kB (Burr and Stewart 2021), although this cross talk has also not been illustrated in breast cancer. Here, we observed genes of RAP1 signaling and AGE-RAGE functioning as a cohesive unit while also being correlated with NF-kB activity. The combined enrichment of AGE-RAGE, HIF1, and RAP1 signaling is of particular interest because it implies a novel interaction between these three processes, common to all our cell lines, in a manner that has not been previously described in breast cancer.

We also observed that our GEM of interest is significantly correlated with NF-kB in both clinical samples and cell culture. Other investigators have flagged the direct effect of RAP1 on the cytoskeleton and NF-kB (Mun and Jeon 2012; Zhang et al. 2015), but here we go further, using our unbiased systems approach to link RAP1 signaling with multiple TFs and pathways. Based on known functions of RAP1, along with the functions of pathways that we found interact with it, we hypothesize that the identified transcriptomic unit is key in relaying information from a cell's physical environment to modulate and maintain the cancer stem cell niche (Roy Choudhury et al. 2019).

Previous studies have established a connection between the NF-kB signaling pathway and regulation of cell shape in breast cancer (Sero et al. 2015; Sailem and Bakal 2017). Our findings also illustrate the significance of this pathway in the regulation of cell morphology, with multiple NF-kB regulators and transcriptional coactivators being flagged in our results. Some morphology-correlated GEMs were significantly differentially expressed between cell-shape subtypes with the *ARNT* KO module being significantly up-regulated in basal-like cell shapes relative to luminal. We also found this GEM to have the highest total correlation with all of the morphological features, indicating a strong association with cell shape. By studying terms enriched in this module from the Enrichr library, we found “TNF-alpha signaling via NF-kB” to be enriched as well as genes down-regulated during AHR nuclear translocator (*ARNT*) shRNA KO. Signaling by TNF is able to activate NF-kB, a TF known to control the expression of many EMT related genes (Pires et al. 2017), which has shown to be more sensitive to TNF stimulation in mesenchymal-like cellular morphologies than epithelial. This was hypothesized to generate a negative feedback that reinforces a metastatic phenotype of breast cancer cells (Sero et al. 2015). Here we observed also that an *ARNT* KO/TNF module is up-regulated in basal-like cell lines, consistent with these findings. ARNT is a protein shown to be involved in regulating tumor growth and angiogenesis along with its binding partner aryl hydrocarbon receptor (AHR) (Huang et al. 2015). Previous studies have also shown its ability to modulate NF-kB signaling with the activated form possibly interfering with the action of activated RELA (Øvrevik et al. 2014). Our findings that the up-regulation of a GEM that is associated with *ARNT* knockdown further give credence to NF-kB being positively regulated in mesenchymal-like cell morphologies. Furthermore, the results of our network propagation yielded activators and transcriptional coactivators of NF-kB (IKBKB [Teo et al. 2010], NR0B2 [Zou et al. 2015], and CREBBP [Bhatt and Ghosh 2014]). These findings indicate that NF-kB is modulated by both phosphorylation (through stimulation by TNF), spatial-temporal location (through RAP1), and transcriptional coactivation (through NR0B2 and CREBBP) in breast cancer in a shape-dependent manner.

Aside from the biological findings of this study, we illustrate an approach for network analysis of a specific course-grained phenotype through expression, a notoriously poor (if cheap and widely available) proxy for gauging intracellular signaling (Piran et al. 2020). In contrast to existing methods that use gene expression as a direct proxy for signaling (Guan et al. 2012; Ben-Hamo et al. 2014; Soul et al. 2015; Padi and Quackenbush 2018), our approach infers TF activities from the expression data and uses these as an anchor to infer upstream signaling networks relevant to the regulation of our phenotypes. TF activities can represent the outcome of a signal transduction process compared with the expression profiles and are thus a better proxy for cell signaling activities of the cell (Szalai and Saez-Rodriguez 2020). Such an approach has been previously used, for example, by the tool CARNIVAL (Liu et al. 2019). However, this and other available tools neglect the propensity for the transcriptome to be regulated in a highly context-specific and modular structure (Kitano 2002; Sharma and Petsalaki 2019). Moreover, their reliance on annotated pathways to describe cell signaling undermines their ability to spot novel functional units specific to a given phenotype. Here, using context-specific GEMs, we produced a network connecting the genes of interest from diverse analyses and used a network propagation algorithm to further focus on signaling proteins of novel interest. Although there inevitably remains a level of bias stemming from the TF regulon and pathway annotations, our bottom-up approach seeks to identify unbiased latent modular structures within transcriptomic data first. This puts the emphasis on data-driven GEMs rather than on literature-derived regulons and pathways. This approach takes an important step toward reducing the bias associated with previously annotated pathways and allows the identification of important regulatory units and their function with respect to cell shape from a system biology point of view. Our network approach allows us to map the interface between two graphically presented systems in the cell: the transcriptome and intracellular signaling. Both can be easily combined with complex, multivariate phenotypic data, which here has revealed a clearer picture of how signaling regulates cell morphology in breast cancer.

The interoperability of this approach is obvious, with any number of continuous variables measured with gene expression able to be correlated with module eigengenes using WGCNA. Here, we used OmniPath as a base network, but other network-based representations of the cellular environment can be used based on the appropriate context. Thus, our method represents a data-driven, network-based approach compatible with many different multiscale phenotypes that are driven by intracellular signaling. Overall, our unbiased network-based method highlights potential “missing links” between sensing extracellular cues and transcriptional programs that help maintain the cancer stem cell niche and ultimately push breast cancer cells into EMT and metastasis. These represent starting points for further experimental studies to understand and therapeutically target the links between cell shape, cell signaling, and gene regulation in the context of breast cancer.

## Methods

### WGCNA analysis

Using weighted correlation network analysis, we performed coexpression module identification using the R package WGCNA (Langfelder and Horvath 2008). We used bulk RNA-seq data from ArrayExpress (in FPKM; E-MTAB-2770 and E-MTAB-2706) acquired from commonly used cancer cell lines of various cancer types and with the alignment performed to the NCBI Human Reference Genome GRCh38 (Papatheodorou et al. 2020). We collated 13 breast cancer and one nontumorigenic cell line for which imaging data were available (BT-474, CAMA-1, T-47D, ZR-75-1, SK-BR-3, MCF-7, HCC1143, HCC1954, HCC70, Hs 578T, JIMT-1, MCF-10A, MDA-MB-157, and MDA-MB-231) (Sero et al. 2015). We acquired representative images of each cell line from Sero et al. (2015; https://datadryad.org/stash/dataset/doi:10.5061/dryad.tc5g4). Cell imaging segmentation was performed using Acapella software (PerkinElmer) with an automated spinning disk confocal microscope. The presented images (Fig. 2) are taken from the above link, stained with DAPI (blue), anti-p65 (Abcam ab16502); Alexa Fluor 488 (anti-rabbit; Invitrogen) (green), and DHE (red). Using Ensembl-BioMart, we filtered genes to only include protein-coding genes (Kinsella et al. 2011) and genes whose FPKM was greater than one, leaving a total of 15,304 genes.

We created a signed, weighted adjacency matrix using log_2_ transformed gene expression values and a soft threshold power (beta) of nine. We translated this adjacency matrix (defined by Equation 1) into a topological overlap matrix (TOM; a measure of similarity), and the corresponding dissimilarity matrix (TOM − 1) was used to identify modules of correlated gene expression (minimum module size of 30). Jackknife cross-validation was used to assess the robustness of the identified modules to the removal of different cell lines from the analysis (Supplemental Fig. S1C), and all showed a high degree of conservation between resampled runs.

(1) $$a_{ij}\;=\;|(1\;+\;cor(x_{i},x_{j}))/2{|}^{\text{β}}.$$

We took morphological variables referring to breast cancer cell lines from Sero et al. (2015), which include 10 significant features shown to be predictive of TF activation. We correlated these features with module eigengenes using Pearson's correlation, and we tested these values for significance by calculating Student asymptotic *P*-values for given correlations. Multiple hypothesis testing was performed using a permutation based procedure, whereby we recalculated the correlation matrix 1000 times with resampled data. We then generated null distributions for each ranked correlation statistic in our matrix and compared them with our real data of the same rank. We include in Supplemental Table S1 confidence intervals of our permutation-based multiple-correction procedure. For the modules that correlated with morphological features (Pearson correlation coefficient 0.5 and Student *P* < 0.05), we identified enriched signaling pathways using the R package Enrichr (Kuleshov et al. 2016) and the signaling database Reactome (Jassal et al. 2020). Reactome was used in preference to other pathway databases because of the more consistent inclusion of TFs within the annotated pathways. Using the database TRRUST v2 (accessed July 1, 2018) (Han et al. 2018), we identified TF regulons that significantly overlap (Fisher's exact test, *P* < 0.1) with the GEM contents. This was performed separately for inhibitory and activatory expression regulons for each TF, with regulatory relationships of unknown sign being used in the significance calculations for both.

We named gene expression clusters using significantly enriched terms identified by the Enrichr analysis (Supplemental Table S3). As some clusters were very obscure, we used the entire Enrichr list of libraries (https://maayanlab.cloud/Enrichr/#stats for full list) with precedence going to the signaling databases of KEGG, Reactome, PANTHER, and Wikipathways (accessed April 1, 2020) (Mi et al. 2013; Kanehisa et al. 2016; Martens et al. 2021). Some modules could not be assigned informative terms and so were named “not annotated” (NA).

### Clustering and differential expression

Using the *k*-means algorithm, we classified the 14 breast cancer cell lines by the median values of each of their shape features (k = 3) (see Supplemental Fig. S2A). We performed differential expression analysis using the R package DESeq2 (Love et al. 2014). We filtered genes so that only protein-coding genes and those with >0.5 counts per million in at least eight cell lines were included. We calculated log_2_ fold changes with the cluster of interest as the numerator and the remaining cell lines acting as a control. Using the R package FGSEA (Korotkevich et al. 2021), we performed gene set enrichment analysis of the differentially regulated proteins using the complete pathways gene set (release April 1, 2020) from MSigDB (Liberzon et al. 2015) and the WGCNA GEMs identified in previous analysis. We calculated TF regulon enrichment using the software DOROTHEA (accessed April 1, 2020) (Holland et al. 2020).

### Network generation

Using a PCSF algorithm, we generated a cell-shape regulatory network implemented through the R package PCSF (Akhmedov et al. 2016). For the prize-carrying nodes to be collected by the PCSF algorithm, we used the TFs significantly regulating the WGCNA modules using TRRUST v2 (*P* < 0.1), the differentially activated TFs identified by DOROTHEA (*P* < 0.1), and the signaling proteins included in the REACTOME pathways that were enriched in TFs identified (*P* < 0.05). We identified these pathways by using the TRRUST TFs identified in the previous steps, as well as ENCODE and ChEA Consensus TFs from ChIP-X (Lachmann et al. 2010), DNA binding preferences from JASPAR (Wasserman and Sandelin 2004; Stormo 2013), TF protein–protein interactions, and TFs from ENCODE ChIP-seq (Euskirchen et al. 2007). Using Enrichr, we identified pathways that were enriched in the identified TFs, and the proteins that were included in these pathways were extracted from Pathway Commons using the R package paxtoolsr (Luna et al. 2016). This was tested for bias to specific pathways by generating pathway-specific null distributions from 1000 resampled GEMs. Distributions of *P*-values for each Reactome pathway were generated, in which failed tests (because of no TF enrichment) were given a *P*-value of one. Results of this were corrected for multiple-hypothesis testing using FDR correction.

The “costs” associated with each edge in the regulatory network were the inverse of the number of sources linked to each regulatory connection scaled between one and zero, such that the more the number of citations for an edge, the lower the cost. For the base network used by the algorithm, we used the comprehensive biological prior knowledge database, OmniPath (accessed May 6, 2020) (Türei et al. 2021), extracted using the R package OmnipathR (Türei et al. 2016). We set each prize for significant TFs or signaling pathways to 100 and used a random variant of the PCSF algorithm with the result being the union of subnetworks obtained on each run (30 iterations) after adding random noise to the edge costs each time (5%). The algorithm also includes a hub-penalization parameter, which we set to 0.005. Other parameters include the tuning of node prices (set to one) and the tuning of trees in the PCSF output (40).

We included the WGCNA modules themselves as supernodes in the network by adding incoming edges from the TFs contained within the regulatory network whose regulomes (as described in TRRUST v2) (Han et al. 2018) significantly overlap (Fisher's exact test; *P* < 0.1) with the gene content of the module in question. We represented the respective cell-shape phenotypes as nodes in a similar fashion by including undirected edges from expression modules and phenotypes in which there was significant correlation (|PCC| > 0.5 and *P* < 0.05) between them. To account for expression modules’ effect on upstream signaling, we added edges from the WGCNA modules back up to proteins that were themselves included within the modules. We set the edge weight of these to 1, such that any predicted activity of the GEM would be translated directly into its constituent signaling proteins and thus account for feedback between cell-shape signaling networks and the context-specific expression modules identified in the first step. We identified enriched terms in the network using the 2016 release of the database PANTHER (Mi et al. 2013) and GSE package Enrichr (Kuleshov et al. 2016).

### Network propagation of functional TFs

We examined the potential effect of significantly activated (FDR < 0.05) and deactivated TFs in different cell line clusters using network propagation in our generated network. We replaced edge weight with the Resnik best-match average (BMA) semantic similarity (Resnik 1995) between the biological process GO terms of the two interacting pairs, with the sign of the interaction being inherited from Omnipath (Türei et al. 2016). We then scaled the semantic similarity edge weights between one and minus one.

We used the differentially activated TFs identified using DOROTHEA (*P* < 0.05) as seeds for a RWR algorithm using the R package diffuseR (available at https://github.com/dirmeier/diffusr). We judged a node to be significantly ranked if its affinity score relative to the inputted seeds was greater than the same node's affinity score with a random walk simulation performed with randomized seeds. We performed this randomized simulation 10,000 times, from which a *P*-value was determined to judge significance (*P* < 0.1). We performed this propagation by RWR for both luminal-like and basal-like morphological clusters on significantly activated and deactivated TFs separately in addition to simulations in which each seed was considered in isolation. We implemented these simulations with a restart probability of 0.95. We generated a graphical representation of the network edges and TFs responsible for the ranking of RAP1 signaling by plotting all the shortest paths between RAP1 and the TFs that caused it to have a non-zero affinity score when each TF was considered in isolation.

### Breast cancer cell morphology following kinase inhibitor treatment

We used single-cell, small-molecule kinase inhibition data from the Harvard Medical School (HMS) LINCS Center (Stathias et al. 2020), which is funded by NIH grants U54 HG006097 and U54 HL127365 (available from https://lincs.hms.harvard.edu/mills-unpubl-2015/, accessed August 1, 2020). This data set is derived from the treatment of six cell lines with a panel of 105 small-molecule kinase inhibitors. They measured textural and morphological variables following treatment by high-throughput image analysis (Haralick et al. 1973; Hamilton et al. 2007). We combined this assay with another data set from HMS-LINCS: a target affinity spectrum (TAS) for compounds in the HMS-LINCS small-molecule library measuring the binding assertions based on dose response affinity constants for particular kinase inhibitors (https://lincs.hms.harvard.edu/db/datasets/20000/, accessed August 1, 2020). Using this data set, we filtered for only molecule-binding target pairs with a binding class of one (representing a K_D_ < 100 nM affinity). Further to this, we removed molecules that had more than five targets with a K_D_ of 100 nM. Consequently, the remaining kinase inhibitors were relatively narrow spectrum, thus simplifying analysis of their phenotypic effect. We expressed these results as batch-specific log fold changes of 10-µm drug treatment relative to the mean of the control set (untreated and DMSO-treated cells). Spearman's rank correlation was calculated between the drug target's network centrality and the absolute log fold change of the morphological variable. We also used the Kolmogorov–Smirnov statistic to assess significance between cell morphology after treatment with drugs targeting kinases inside versus outside our predicted network. This was also repeated on other breast cancer cell lines and using a TRAIL control (Supplemental Table S8).

The morphological data in the kinase inhibition screen were measured using two dyes (DRAQ5 and TMRE), the intensity of which we used to normalize textural features and the measurement of cytoplasmic and nuclear small spots. We reported counts for small-nuclear or cytoplasmic spots as a mean of the individually normalized readings from both dyes. We considered a treatment perturbing our network if at least one of the kinase inhibitors targeted a protein that was represented by a node within the network.

### Quantifying kinase inhibitor influence

We incorporate information from the TAS assay, as well as graph-based properties of kinase inhibitor targets, using the product of the Szymkiewicz–Simpson similarity (measured between the cell-shape network nodes and the drug targets) and the centrality of the targeted nodes in the predicted network with semantic similarity edge weights. The product of these generates, for a given kinase inhibitor, the statistic

(2) $$\sum_{x\in \;K\cap N}PR(x)\times \frac{K\cap^{N}}{\min(\left|K|,\;|N\right|)},$$

where K is the set of kinases an inhibitor is predicted to target, N is the nodes of the network, and the function PR() is the centrality of a particular node in the network as defined by the PageRank algorithm (Brin and Page 1998). This centrality measure has been shown to be effective in prioritizing proteins by relative importance in signaling or protein–protein interaction networks (Iván and Grolmusz 2011). We used this statistic as a measure of a kinase inhibitor's influence on cell shape.

### Analysis of BRCA cell line and TCGA sample RNA-seq data

For the cell lines, we used RNA-seq data from the ArrayExpress (in FPKM; E-MTAB-2770 and E-MTAB-2706) (Papatheodorou et al. 2020). This was analyzed using DESeq2 (Love et al. 2014) per the methodology in the “Clustering and Differential Expression” section. Both TF and module activity were calculated using the algorithm VIPER (Alvarez et al. 2016). For patient data, the results shown here are based on data generated by The Cancer Genome Atlas (TCGA) Research Network (https://www.cancer.gov/tcga, accessed 01/04/21). For computational efficiency, we used gamma-Poisson models to predict differentially expressed genes from our samples using the package glmGamPoi (Ahlmann-Eltze and Huber 2021). We used the sample of interest as the numerator with the remaining tumor samples acting as a control. For quantifying correlation between RAP1 GEM and different TFs, we removed samples with insignificant activation of either the TF in question or RAP1 GEM (FDR adjusted *P*-value < 0.05). Correlation was quantified using the Kendall rank correlation coefficient. Differences in distributions of morphological subtypes was quantified by the Kolmogorov–Smirnov test.

### Maximum-flow network analysis

For maximum-flow calculations, we used the Resnik BMA semantic similarity (Resnik 1995) as the maximum “carrying capacity” of an edge in the network. To visualize the optimized solution (as implemented by the R package igraph) (Csardi and Nepusz 2006), we selected only those edges in the 99th percentile of the flow-carrying edges in the network. Visualization was performed using the software Cytoscape (Shannon et al. 2003). Maximum flow was performed with the R package igraph (Csardi and Nepusz 2006).

### Quantification and statistical analysis

Statistical tests were performed in base R (R Core Team 2021) unless otherwise mentioned in the Methods, and *P*-value cut-offs are shown in parentheses after reporting an effect as significant. A weighted Pearson's correlation with *t*-test for significance was used to correlate eigengenes and cell-shape features using the RNA package WGCNA. We used a one-way ANOVA test for comparing the means of the shape variables among the identified three cell line clusters (*n* = 75,653) and a Tukey honest significant difference test to perform multiple-pairwise comparison among the means of the groups. The same tests were performed on the differences in 10 cell-shape variables when Hs 578T was treated with 37 kinase inhibitors (*n* = 23,128). A Fisher's exact test was used to test significance of overlap between TRRUST regulons and identified GEMs (for size of the overlap, see Supplemental Table S4).

Enrichment of gene sets was performed by Enrichr, an enrichment library that uses a hypergeometric test to identify significantly enriched terms in a gene list. This tool (described by Chen et al. 2013) calculates a score combining the Fisher's exact test *P*-value of the enrichment with the *z*-score deviation from the expected rank. The preranked gene set enrichment algorithm FGSEA was used for the identification of enriched terms in the differentially expressed genes, allowing for accurate estimation of arbitrarily low *P*-values that occur in expression data sets.

Spearman rank correlation was used to measure the strength of the association between target network centrality and the measured effect of its perturbation by inhibition. Spearman was chosen because the centrality (combined with Szymkiewicz–Simpson) according to Equation 2 does not follow an exact normal distribution. Kendall rank correlation coefficient was used when calculating the correlation between TF activity and RAP1 GEM activity because confidence intervals for Spearman's r_S_ are less reliable and less interpretable than confidence intervals for Kendall's tau parameters. When trying to distinguish between many correlations of similar quality, this becomes more important. FDR adjustment for multiple testing correction was always used when multiple tests were performed in the same analysis.

Kolmogorov–Smirnov test was used to measure differences in distributions of clinically assigned tumor morphologies. This was because clinical groupings are mixed (i.e., infiltrating duct and lobular carcinoma), and others are characterized by an absence of features over their presence. This means that the assumption of normality required for a *t*-test is not fulfilled.

For differential expression analysis, the DESeq2 R package (Love et al. 2014) was used. DESeq2 fits negative binomial generalized linear models for each gene and uses the Wald test for significance testing. The package then automatically detects count outliers using Cooks's distance and removes these genes from analysis.

Significance was determined for RWR network propagation by randomizing seed nodes (preserving their values) 10,000 times and selecting only the nonseed nodes that were significantly ranked relative to the randomized simulations (*P* < 0.1). Figures were presented using ggplot (Wickham 2009).

### Software availability

The complete R scripts and data used for this methodology are available as Supplemental Code and at GitLab (https://gitlab.ebi.ac.uk/petsalakilab/phenotype_networks).

## Supplementary Material

Supplemental Material
