# Supplementary material for: Identification of phenotype-specific networks from paired gene expression–cell shape imaging data
Source: Genome Res. 2022 Apr;32(4):750–65. doi: 10.1101/gr.276059.121 (PMC8997347; doi:10.1101/gr.276059.121)
Supplement: Supplemental Material [file supp_32_4_750__DC1.html]

Identification of phenotype-specific networks from paired gene expression–cell shape imaging data — Supplemental Material 

# Identification of phenotype-specific networks from paired gene expression–cell shape imaging data

## Supplemental Material

- Supplemental\_Figures.pdf
- Supplemental\_Table\_S1.xlsx
- Supplemental\_Table\_S2.xlsx
- Supplemental\_Table\_S3.xlsx
- Supplemental\_Table\_S4.xlsx
- Supplemental\_Table\_S5.xlsx
- Supplemental\_Table\_S6.xlsx
- Supplemental\_Table\_S7.xlsx
- Supplemental\_Table\_S8.xlsx
- Supplemental\_Table\_S9.xlsx
- Supplemental\_Code.zip
