# Supplementary material for: Identification of phenotype-specific networks from paired gene expression–cell shape imaging data
Source: Genome Res. 2022 Apr;32(4):750–65. doi: 10.1101/gr.276059.121 (PMC8997347; doi:10.1101/gr.276059.121)
Supplement: Supplemental Material [file supp_gr.276059.121_Supplemental_Figures_.pdf]

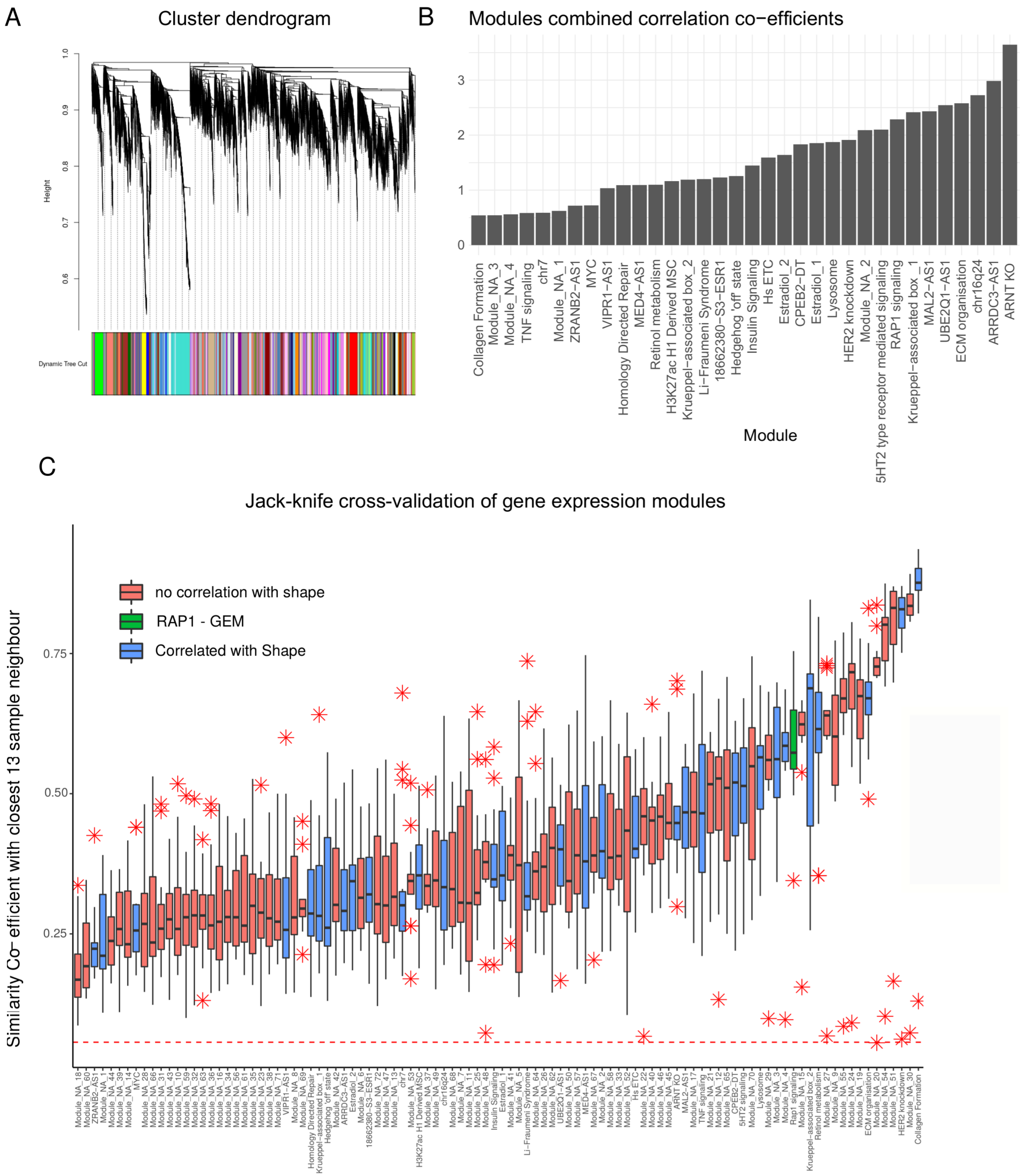

Supplemental Figure S1A - Gene expression dendrogram showing the hierarchical arrangement of gene expression in breast cancer cell lines. Derived gene expression modules are shown along the bottom. **B –** Bar chart showing the sum of the absolute values of Pearson's correlation coefficient (PCC) for the significantly correlated ($P<0.05$) module – feature associations. **C -** Bar plot showing the Overlap Coefficient (y axis) of the closest analogue to a 14-cell line module (x axis) within our 13-cell line jack-knifed set of modules. The dotted red line along the bottom illustrates the 99th percentile of the similarity exhibited by 1,000 randomised modules of the same lengths. Modules are coloured based on whether they are correlated with cell shape (blue), not correlated at all (red) and the RAP1 module is colour green.

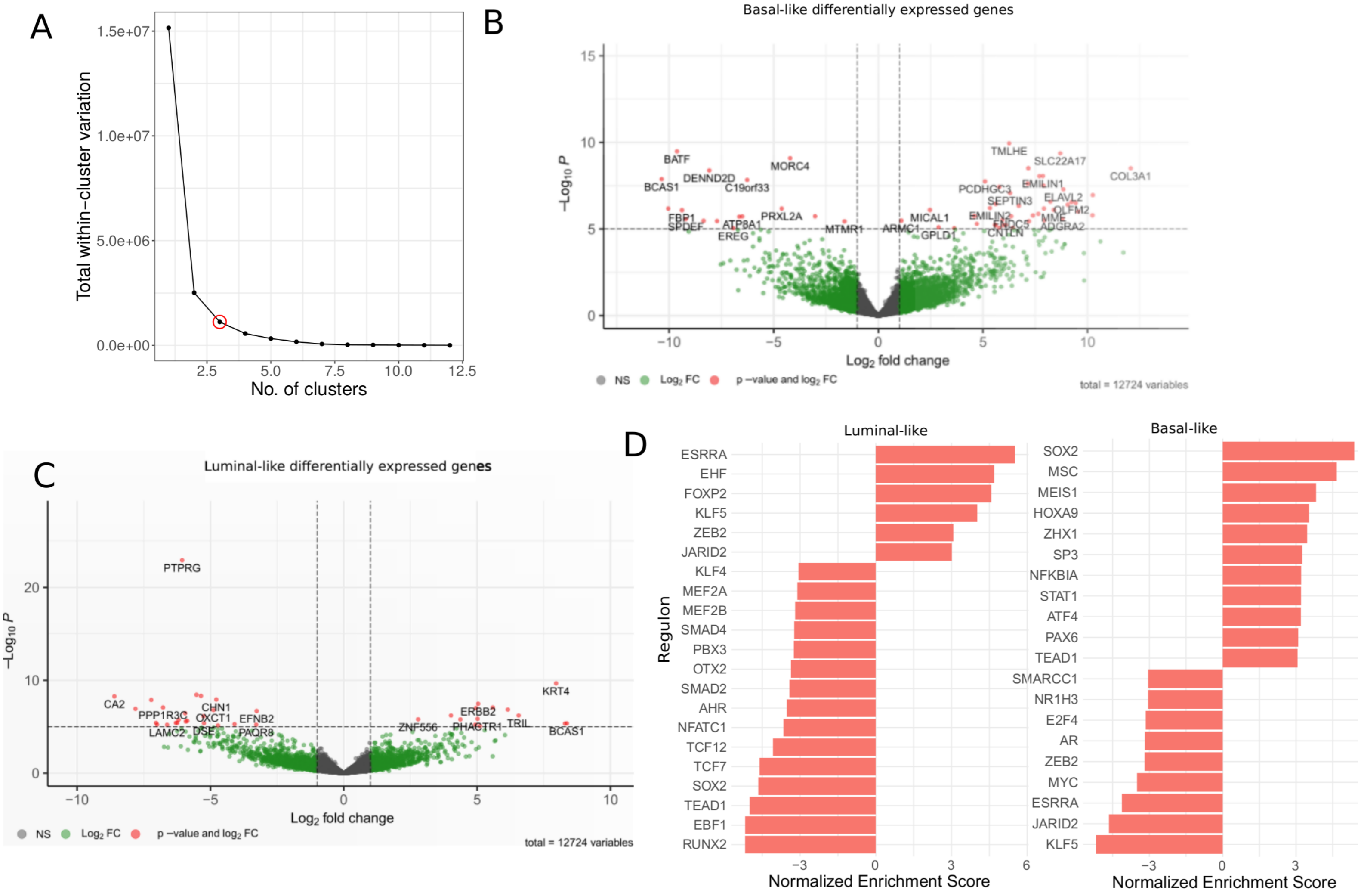

Supplemental Figure S2A - Elbow plot illustrating the diminishing return of decreasing total within-cluster variation of cell shape groups as the number of clusters is increased. A red circle shows the selected statistic for k (3). **B-C -** Volcano plots showing significantly differentially expressed genes for the basal-like (B) and luminal-like (C) morphological clusters. The x-axis shows the log fold change and the the y axis shows the significance (-Log_{10}(P)). A dotted horizontal line indicates a significance cut-off of $P<1x10^{-5}$. **D.** TF activity for morphological clusters C and B (luminal-like and basal-like respectively) as derived from RNA-seq

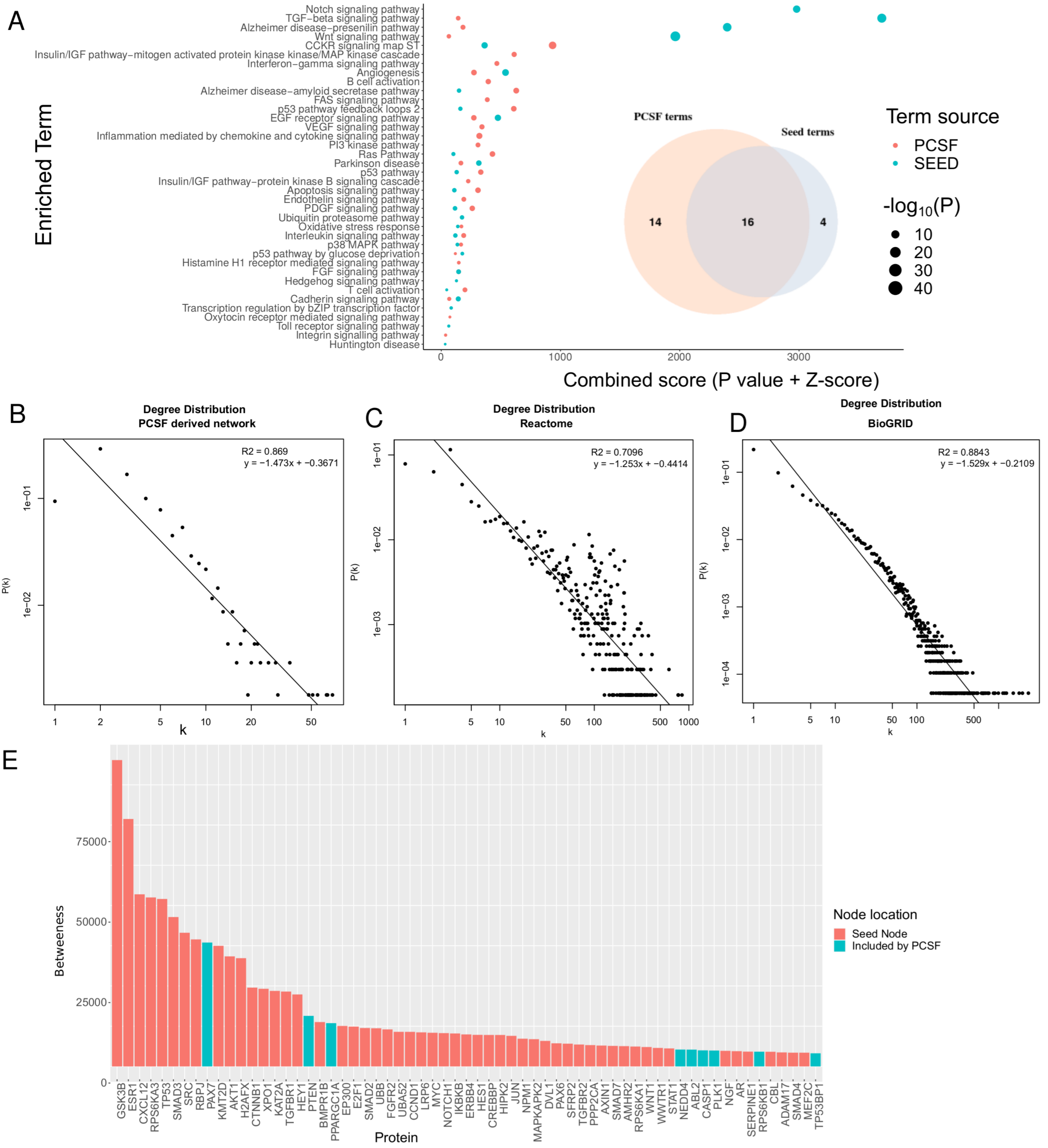

Supplemental Figure S3A - Gene set enrichment (Panther DB 2016) of original seed nodes and the nodes included by PCSF with the dot size indicating the level of significance (-Log10P) of the term enrichment. Blue nodes represent enrichments from proteins that were used as inputs for the PCSF algorithm and red nodes are those that were included because of it. Inset is a Venn diagram showing the overlap in enriched terms between the seed nodes and the terms included by PCSF. **B-D** - Plots showing the scale degree free distribution of our PCSF derived network (A), consistent with the same plots from Reactome (B) and BioGRID (C) on a log-log scale. The x-axis shows the number of connections for a given vertex (degree - shown by k), while the y axis represents the proportion of vertices in the network having a degree of k. The degree exponent is shown as the gradient of the line of best fit. **E -** Bar chart showing the betweenness centrality of nodes in the derived regulatory network, with centrality on the y axis and the nodes (representing proteins) on the x axis. Nodes are coloured red if they were original seed nodes used in the PCSF and blue if they were included by the algorithm. Only those nodes with centrality > 4,000 are shown.

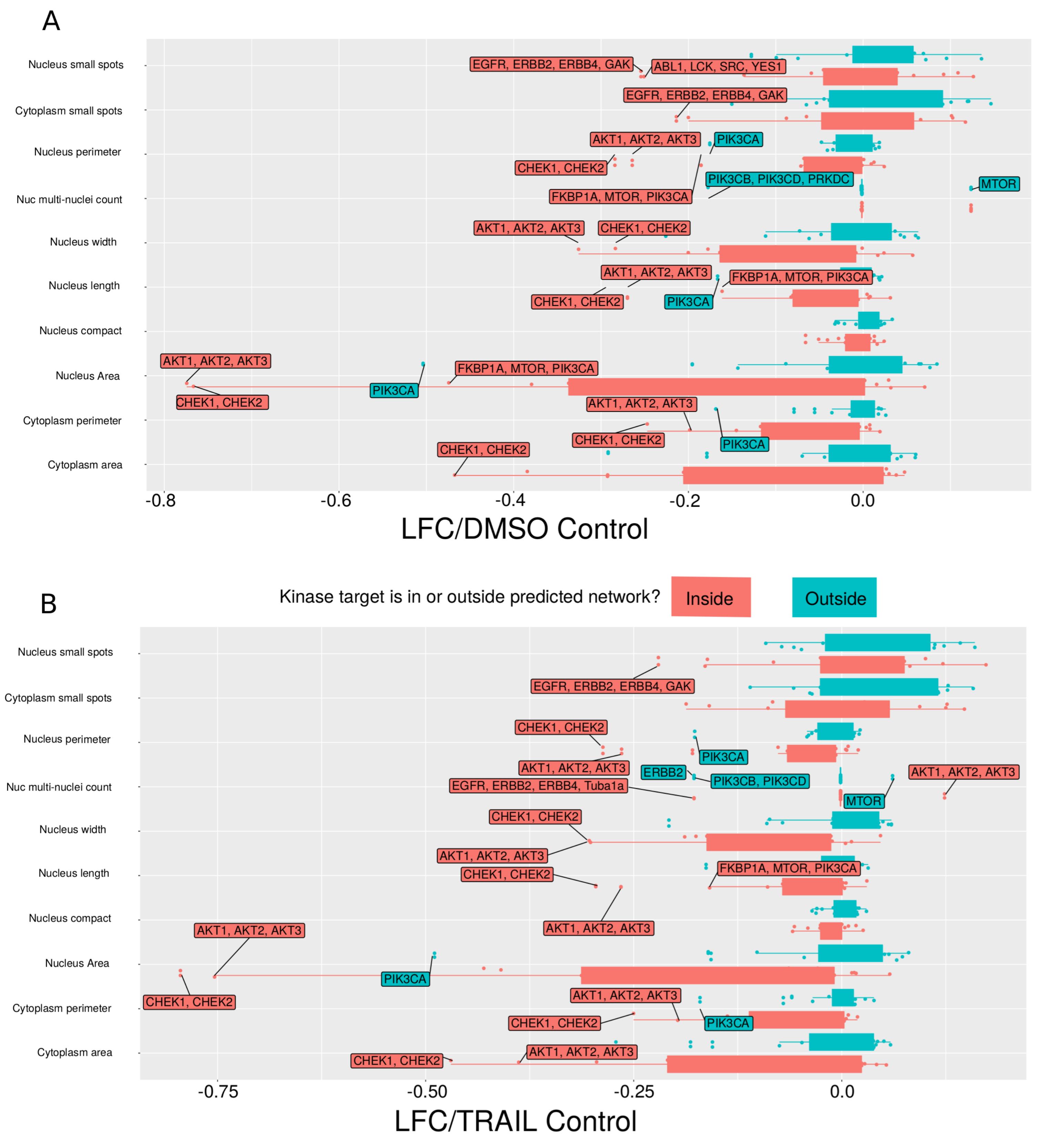

Supplemental Figure S4 - Box plots showing the log fold changes of cell shape variables after treatment with a drug relative to a DMSO control (A) and TRAIL control (B). The kinase targets of the drugs are shown as labels for the outliers (IQR method), with inhibitors targeting drugs within the predicted network coloured red and those targeting kinases not included in our network coloured blue.

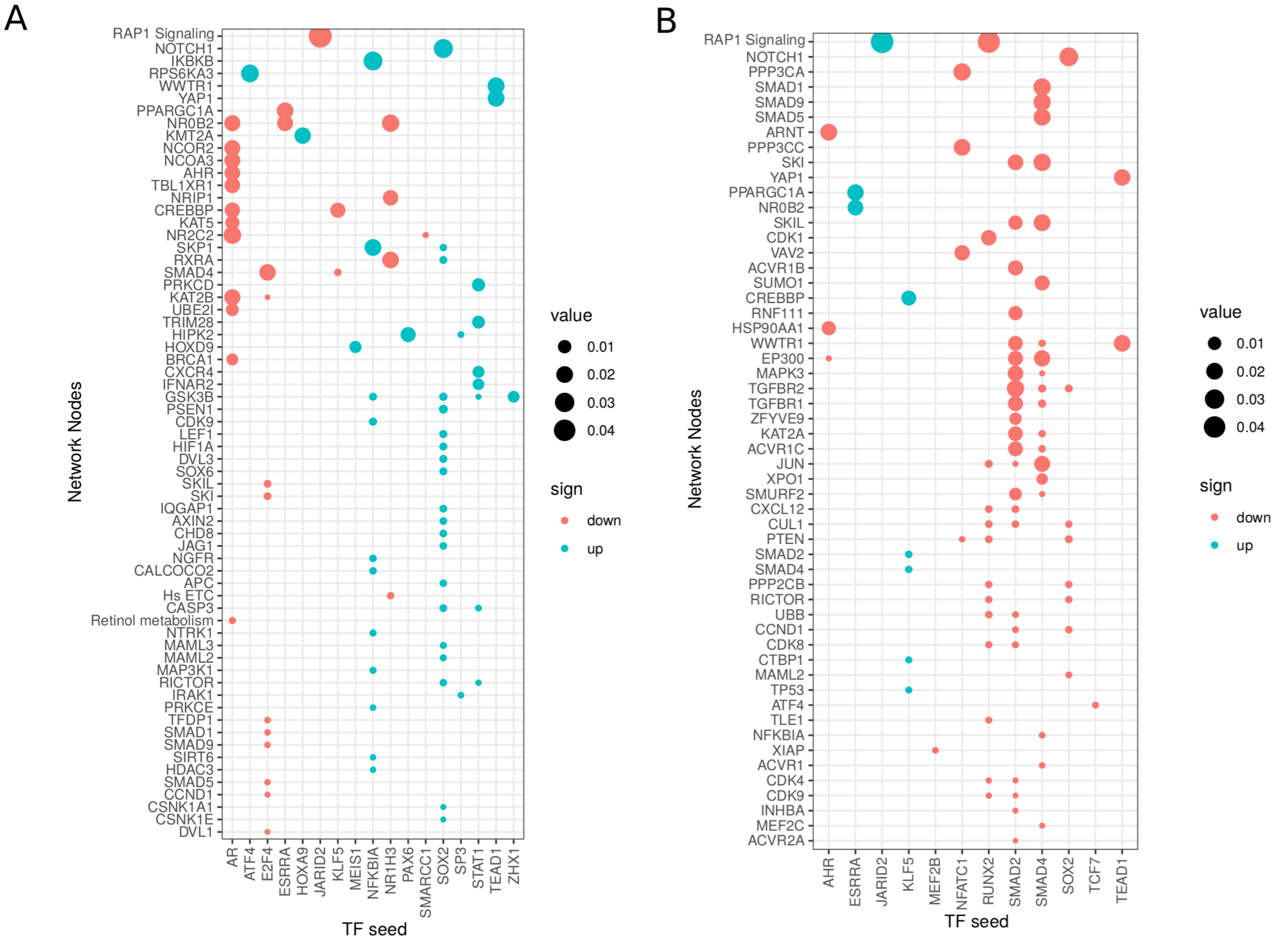

Supplemental Figure S5A - Dot plot showing network propagation in predicted cell shape networks from activated and inactivated transcription factors in basal-like cell lines, performed one seed node at a time. The y axis shows different nodes in the network, with red stars indicating super-nodes (gene expression modules). The x axis shows the TFs used as seed nodes. The size of the dot indicates the steady state probability over the graph imposed by the starting seeds and the colour shows whether or not the TF source was predicted to be activated or deactivated from the gene expression data. **B -** Dot Plot illustrating the network propagation for luminal-like cell lines.

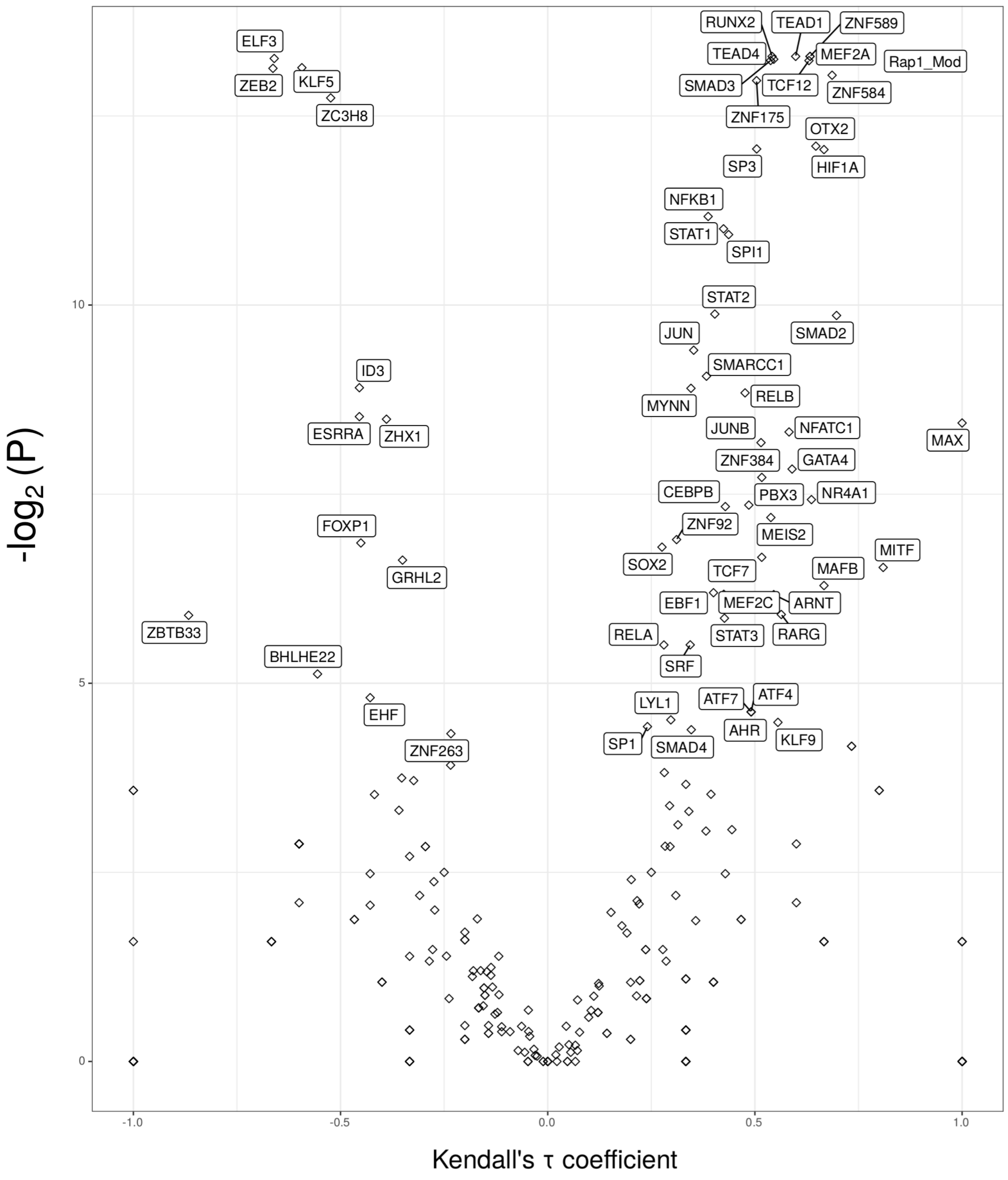

Supplemental Figure S6 - Volcano plot illustrating the correlation (Kendall's rank correlation) between activity of RAP1 gene expression module and transcription factor activity in 78 breast cancer cell lines. Kendall's tau coefficient along the x axis and -log_2 (FDR adjusted P) along the y axis.
